# Adult attachment insecurity and responses to prolonged severe occupational stress in hospital workers during the COVID-19 pandemic

**DOI:** 10.1080/21642850.2022.2123806

**Published:** 2022-09-15

**Authors:** Robert G. Maunder, Natalie D. Heeney, Jonathan J. Hunter

**Affiliations:** a Sinai Health and Department of Psychiatry, University of Toronto, Toronto, Canada; b Sinai Health, Toronto, Canada

**Keywords:** Adult attachment, stress response, coping, resilience, occupational burnout

## Abstract

**Background:**

The stress response includes appraisal of the threat and one’s resources, coping (including interpersonal interactions), distress, and recovery. Relationships between patterns of adult attachment and stress response have received little study in the context of prolonged, severe occupational stress, limiting knowledge about how attachment patterns contribute to occupational burnout and recovery.

**Aim:**

This study aimed to assess the relationship of adult attachment to aspects of the stress response over time in hospital workers during a pandemic.

**Methods:**

This study included 538 hospital workers within a general and a rehabilitation hospital in Toronto, Canada between September 2020 and November 2021. Half, selected at random, completed validated measures of adult attachment, resilience, self-efficacy, coping, interpersonal problems, and various stress outcomes. Attachment insecurity severity was calculated as the vector addition of attachment anxiety and attachment avoidance. Correlations between these measures were determined at individual time-points and temporal patterns of adverse outcomes using repeated-measures ANOVA.

**Results:**

All correlations between measures of attachment and resilience or self-efficacy were significant and moderately strong (*r* = .30–.48), while most correlations with coping strategies were weak (<.20). Attachment avoidance was more strongly correlated with interpersonal problems related to being cold, whereas attachment anxiety was more strongly correlated with problems related to being intrusive, overly-nurturant, exploitable and non-assertive. Attachment insecurity severity was moderately correlated with every dimension of interpersonal problems. A significant main effect of each attachment measure on each stress outcome was found (effects sizes: .18–.26). Attachment insecurity severity was significantly associated with outcome X time interactions for burnout, consistent with greater resilience for those with lower attachment insecurity.

**Conclusions:**

Severity of insecure attachment was correlated with each measure of self-appraisal, interpersonal problems, and all measured stress outcomes. Severity of attachment insecurity performed well as a summary attachment measure. Greater security is associated with patterns of recovery that indicate resilience.

## Introduction

The attachment system is closely related to regulation of responses to perceived threats throughout life (Engel & Gunnar, 2020). In health research, enduring patterns of adult attachment are usually assessed and measured via self-appraisal of preferences and attitudes within close relationships (Ravitz et al., 2010). In this framework, insecure attachment consists of two dimensions, attachment anxiety and attachment avoidance, which can be understood as two relational strategies for managing perceived threats in the absence of an available, responsive attachment figure (Mikulincer & Shaver, 2016). Attachment anxiety refers to hyper-activating strategies, such as help-seeking and expressing distress, as well as hypervigilance regarding threats. Attachment avoidance refers to relative de-activation of these strategies. These two dimensions of attachment are theoretically independent of each other and can be considered to create a two-dimensional space in which an individual’s attachment style is represented by a position (x,y) in which x is their degree of attachment anxiety and y is their degree of attachment avoidance (Maunder & Hunter, 2012).

Mikulincer and Shaver’s 2016 review (Mikulincer & Shaver, 2016) summarizes the evidence linking adult attachment to elements of the stress response: appraisal of threats, appraisals of one’s own resources to respond to threats, choices of coping behavior, the interpersonal context of the threat, and outcomes of this exposure. Greater attachment security (i.e. lower insecurity) is associated with appraising stressful events as less threatening (Mikulincer & Florian, 1995; Taubman-Ben-Ari et al., 2009) and appraising oneself as more able to cope effectively with them (Klohnen et al., 2005; Wei et al., 2003), although the relationships of these appraisals to specific dimensions of attachment insecurity are inconsistent (Mikulincer & Shaver, 2016). In contrast, some coping strategies have been more consistently linked to specific dimensions of attachment insecurity. Mikulincer and Shaver’s review finds: (a) coping strategies which are consistently emphasized by those with greater attachment avoidance (distancing strategies including denial, repression, distraction, cognitive or emotional disengagement, and resignation) (Holmberg et al., 2011; Shapiro & Levendosky, 1999); (b) coping strategies that are consistently emphasized by those with greater attachment anxiety (emotion-focused strategies such as wishful thinking, self-blame, worry and rumination), (Consedine et al., 2013; Mikulincer & Florian, 1999); and (c) coping strategies for which the evidence for an association with attachment security and orientation is inconsistent (problem-focused coping, such as planning, problem-solving, and positive reframing). Some studies find that emotion-focused coping strategies that are typically associated with attachment anxiety may also be reported by those with attachment avoidance, which Mikuliner and Shaver interpret as the result of a failure of deactivating strategies under conditions of high threat. Studies of substance use, which can be understood as a maladaptive coping strategy for circumstances of high stress, are associated with both attachment avoidance and attachment anxiety (i.e. with attachment insecurity in general) (Meredith et al., 2020; Roy et al., 2021).

Interpersonal problems are highly relevant to managing environmental stress because interpersonal difficulties are a potential source of stress, whereas interpersonal support may act as a buffer (Roy et al., 2021; Szkody et al., 2021). Attachment avoidance is associated with interpersonal problems that result from excessive distancing strategies, whereas attachment anxiety is associated with interpersonal problems related to excessively seeking closeness. In the circumplex model of interpersonal problems (Kiesler, 1996), problems related to distancing are being cold, vindictive (i.e. cold + domineering), and socially avoidant (i.e. cold + non-assertive). Interpersonal problems related to excessively seeking closeness are being overly nurturant, intrusive (i.e. overly nurturant + domineering), and exploitable (i.e. overly nurturant + non-assertive).

**Figure 1.**
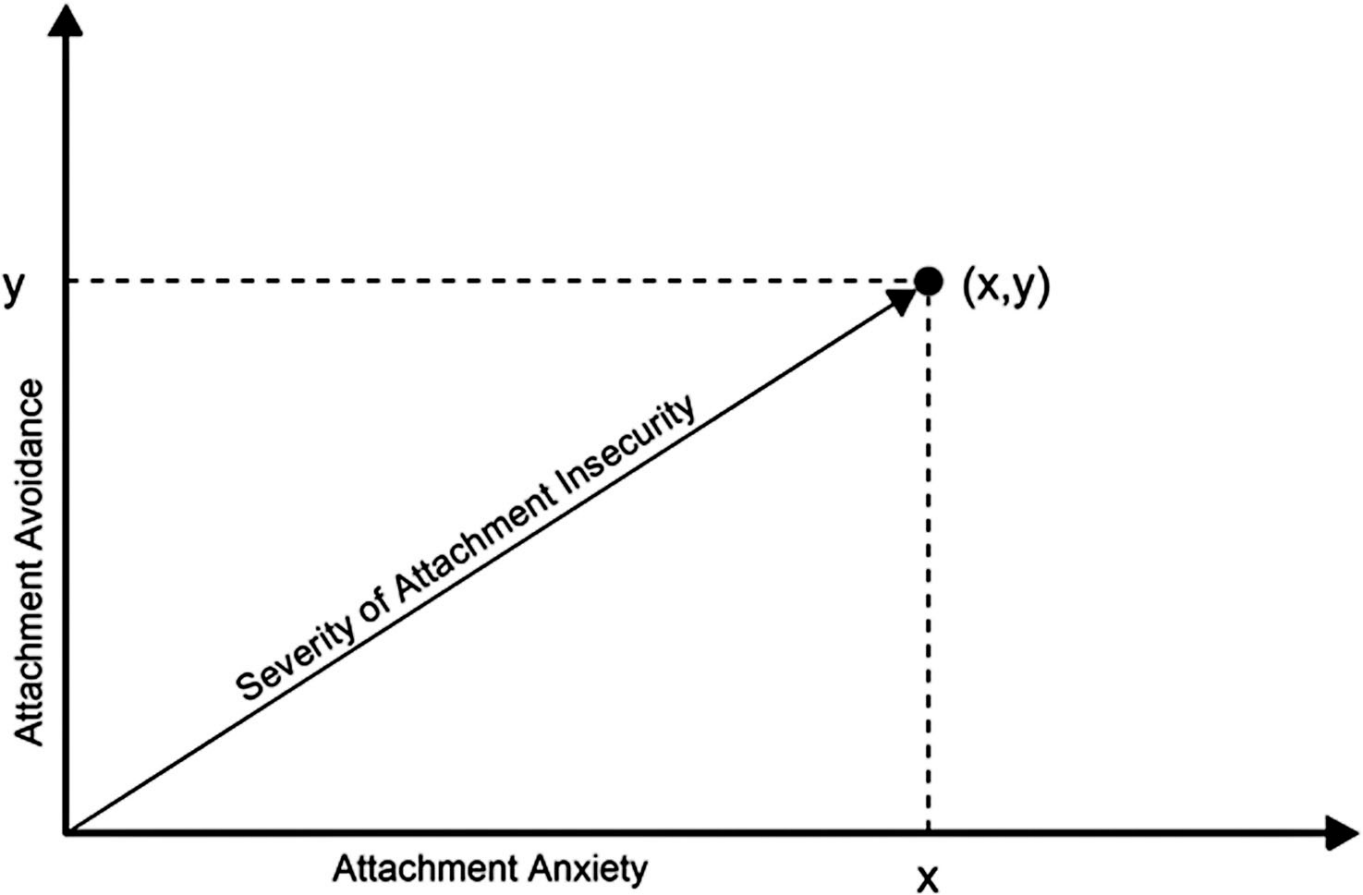
Severity of attachment insecurity conceptualized as the vector addition of attachment anxiety and attachment avoidance.

Most research on adult attachment as a determinant of stress responses studies the two dimensions of insecurity separately, and therefore, emphasizes the differential impact of hyper-activating and deactivating attachment strategies on stress responses, rather than the overall impact of the severity of insecurity. The potential under-emphasis on overall severity of attachment insecurity is important, because high attachment anxiety, high attachment avoidance and high overall insecurity call for different mitigation strategies (see, for example, chapters 12, 13 and 14 in Maunder and Hunter, 2015). This emphasis may, in part, be the result of validated instruments which measure attachment avoidance and attachment anxiety directly, but do not directly measure severity of insecurity. We have argued, based on clinical experience, that overall severity of attachment insecurity may be more important than the specific interpersonal strategies one uses to manage it (i.e. specific dimensions of insecurity) for some health outcomes (Maunder & Hunter, 2012), but testing this hypothesis requires a method to measure of overall severity of attachment insecurity. Figure 1 illustrates that when dimensions of attachment are conceptualized as the axes of a two-dimensional space, severity of insecurity can be calculated as the length of the vector that extends from the origin to the point (x,y) that describes an individual’s attachment style, providing a quantitative measure suitable for analysis.

In this study, we analyze attachment anxiety, attachment avoidance, and severity of attachment insecurity as they relate to appraisal of ones’ resources for coping with threat, coping behaviors, interpersonal problems, and stress outcomes over time. We conduct this study in the context of a prolonged and severe occupational exposure, namely working in a hospital during the COVID-19 pandemic. Our primary aim is to distinguish between aspects of the stress response that are most strongly correlated to a particular dimension of attachment insecurity and aspects of the stress response for which severity of attachment insecurity in general is predictive.

With respect to this primary aim, the review of the literature provided above suggests the following specific hypotheses: (i) Self-appraisal of one’s personal resources to respond to threat will not differ between dimensions of attachment insecurity and that their correlation to severity of attachment insecurity will be at least as strong; (ii) regarding coping strategies, attachment avoidance will be more strongly associated than attachment anxiety with distancing strategies (self-distraction, denial, behavioral disengagement, acceptance), attachment anxiety will be more strongly associated than attachment avoidance with support-seeking and emotion-focused coping (seeking emotional support, seeking instrumental support, self-blame, and venting), whereas coping via substance use will not differ in its association to attachment avoidance and attachment anxiety; (iii) attachment avoidance will be more strongly associated than attachment anxiety with interpersonal problems related to excessive distancing (vindictive, cold, socially inhibited), attachment anxiety will be more strongly correlated than attachment avoidance with interpersonal problems related to excessively seeking closeness (overly-nurturant, exploitable, intrusive), and severity of insecurity will be most strongly associated with the mean severity of all interpersonal problems; and (iv) outcomes of severe and prolonged occupational stress (burnout, psychological distress, and posttraumatic symptoms) will not differ between dimensions of attachment insecurity and that their correlation to severity of attachment insecurity will be at least as strong.

Much more is known about cross-sectional associations between aspects of adult attachment and stress responses, than about longitudinal patterns of stress response and recovery. In particular, relatively few studies (for example, Mikulincer & Florian, 1995; Tosone et al., 2015) have investigated the relationship between attachment insecurity and the impact of severe and persistent environmental threats. Therefore, a secondary aim is to explore the relationship between attachment insecurity and longitudinal trends in stress outcomes during the prolonged real-world stressor of working in healthcare during the COVID-19 pandemic.

## Methods

A staff survey of psychological well-being and related measures during the COVID-19 pandemic was conducted at two sites of Sinai Health (a general hospital and a rehabilitation hospital) in Toronto, Canada at five time-points (T1: Sept 21–Nov 15, 2020; T2: Jan 25–Feb 15 2021; T3: Apr 26–May 16 2021; T4: Jul 26–Aug 15 2021; T5: Oct 25–Nov 14 2021). The survey methods have been described previously (Maunder et al., 2021). Inclusion criteria were being a student or worker at the hospital in the summer of 2020 (including all hospital employees, physicians, learners, volunteers, retail employees, and contractors, whether or not they had regular patient contact). Respondents less than eighteen years old were excluded. The survey was advertised through posters, hospital emails, and local managers. All qualifying, consenting respondents were included. The participation rate cannot be calculated because its denominator (i.e. the number of staff who were sufficiently informed to make a decision to participate or not) is unknown. As an upper limit, the entire salaried workforce who might have participated was approximately 6000. All surveys were completed online using software (Alchemer, Louiseville, CO) that is compliant with privacy legislation in the study’s jurisdiction.

Of 884 respondents who provided consent in a pre-survey recruitment phase, 538 (61.0%) completed a T1 survey to form the cohort for further follow-up. The participation rate at each time point (calculating the numerator as the number of surveys returned that included a valid measure of emotional exhaustion, psychological distress, or both) was: T2 *N* = 485 (90% of T1 cohort), T3 *N* = 424 (79%), T4 *N* = 409 (76%), T5 *N* = 395 (73%). A gift card (about US$15 value) was provided for each completed survey. The study was approved by the Sinai Health Research Ethics Board (REB 20-0084-E).

In order to reduce the risk that survey burden would diminish continued participation over time, instruments that were crucial to the primary purpose of the study (to be reported elsewhere), were included in surveys for every participant, whereas instruments measuring constructs of additional interest were added to the surveys of a randomly chosen 50% of subjects, including different measures at different time points to reduce survey burden. Using an online randomizer in blocks of 8, participants were randomly assigned in a 1:1 ratio to one of two cohorts that received short surveys (‘express’) or longer surveys (‘enriched’). The express survey included measures of psychological distress, emotional exhaustion (a subscale of burnout), and pandemic self-efficacy at every time point. The enriched survey included, in addition, extra measures which varied by time point, including at different time-points the depersonalization and personal accomplishment subscales of burnout, resilience characteristics, coping behavior, interpersonal problems, adult attachment, and posttraumatic symptoms. The participation rate for respondents randomly assigned to the enriched survey was T1 *N* = 281, T2 *N* = 255 (91%), T3 *N* = 218 (78%), T4 *N* = 210 (75%), T5 *N* = 203 (72%).

Adult attachment insecurity was measured with a 16-item modification of the Experiences in Close Relationships-Revised (Sibley et al., 2005), the ECR-M16 (Lo et al., 2009). Attachment anxiety and attachment avoidance are scored as the mean of eight items each, on continuous scales from 1 to 7. The original validation study demonstrated good internal consistency and test–retest reliability (Lo et al., 2009). Attachment was measured at T4. Cronbach’s alpha for attachment anxiety was .88 and for attachment avoidance was .80. We calculated severity of attachment insecurity (the hypotenuse of a right angled triangle with sides consisting of attachment anxiety and attachment avoidance as per Figure 1) using the Pythagorean Theorem: severity of insecurity = √(attachment anxiety^2^ + attachment avoidance^2^).

Resilience characteristics were measured with the 20-item Resilience at Work scale (Winwood et al., 2013). Subscales measure living authentically (3 items), finding your calling (4 items), maintaining perspective (3 items), managing stress (4 items), interacting cooperatively (2 items), staying healthy (2 items), and building networks (2 items). Each item is scored on a 7-point Likert scale (0-6). For this study we used the overall score (sum of all items) converted to a score from 0 to 100 in which higher numbers indicate greater resilience. Resilience characteristics were measured at T1. Cronbach’s alpha was .87.

Pandemic self-efficacy was measured with an instrument first developed for the 2009 H1N1 pandemic (Maunder et al., 2010). It has 23 items probing confidence in one’s ability to meet pandemic-related challenges (e.g. ‘trust in the infection control procedures that are in place’, ‘perform duties that are outside your usual job’) scored on a 5-point scale (1–5), yielding a score from 23 to 115. Pandemic self-efficacy at T4 was included in this analysis. Cronbach’s alpha was .95.

Coping behavior was measured with the Brief COPE, a 28-item measure that assesses 14 types of coping activity as the mean of 2 items each scored 0–4 (Carver et al., 1989). Coping was measured at T1. Types of coping measured are listed with the Cronbach’s alphas for each subscale in parentheses: self-distraction (.49), active coping (.76), denial (.79), substance use (.95), seeking emotional support (.70), seeking instrumental support (.78), behavioral disengagement (.66), venting (.53), positive reframing (.66), planning (.68), humor (.83), acceptance (.68), religion (.85), and self-blame (.70). Types of coping with Cronbach’s alpha ≥ .70 were included in analyses (Carver et al., 1989).

Interpersonal problems were measured with the 32-item Inventory of Interpersonal Problems (Barkham et al., 1996). Participants rate on a 5-point scale (0–4) the degree to which they experience interpersonal problems in 8 domains. These domains, which can be plotted onto the interpersonal circumplex (Kiesler, 1996), are listed with the Cronbach’s alphas for each subscale in parentheses: domineering (.76), vindictive (.93), cold (.89), socially avoidant (.84), non-assertive (.88), exploitable (.79), overly-nurturant (.76), and intrusive (.72). The 32-item version has psychometric properties that are similar to the 127-item original scale (Barkham et al., 1996). Interpersonal problems were measured at T2.

Burnout was measured with the Maslach Burnout Inventory (MBI-HSS [MP]), which measures emotional exhaustion (9 items scored from 0 to 6, yielding a score from 0 to 54), depersonalization (5 items scored from 0 to 6, yielding a score from 0 to 30), and diminished personal accomplishment (8 items scored from 0 to 6, yielding a score from 0 to 48) (Maslach et al., 2021). In this cohort Cronbach’s alpha at the 5 time-points was in the following ranges: emotional exhaustion (.94–.95), depersonalization (.87–.92), personal accomplishment (.83–.91).

Psychological distress is comprised of depressive and anxiety symptoms which is measured as a screening test or as a measure of severity of common mental disorders. Psychological distress was measured with the Kessler K6, which has 6 items scored from 0 to 4, yielding a range of 0–24 (Kessler et al., 2002). The K6 strongly discriminates between community cases and non-cases of psychiatric disorders diagnosed by structured interview (Kessler et al., 2002) and has acceptable sensitivity and specificity (Staples et al., 2019). In this cohort Cronbach’s alpha at each of the 5 time-points was .85.

Posttraumatic symptoms were measured with the Impact of Events Scale-Revised (Weiss & Marmar, 1997), a 22-item measure (for DSM-IV) that assesses hyperarousal, avoidance, and intrusion caused by traumatic events. Respondents are asked to identify a stressful life event (in this case specified as ‘working during COVID-19’) and then to rate how much they were bothered by 22 types of difficulty in the past 7 days (each scored from 0 to 4). The Cronbach’s alpha for the full scale at each time-point this measure was used (T1, T3, T5) ranged from .94 to .96.

Participants assigned to the express or enriched surveys were compared with respect to gender, age, job type, and scores on emotional exhaustion and psychological distress using chi-square tests or *T*-tests as appropriate.

In calculating correlations, for stress related variables that were measured at more than one time point, the measurement closest to T4 (at which attachment was measured) was selected. Linear associations between attachment dimensions (attachment anxiety, attachment avoidance, and severity of insecurity) and other variables were calculated using Spearman’s rank-order correlations because not all variables were normally distributed. To determine if the correlation between a given stress response variable and attachment anxiety vs. attachment avoidance differed significantly, correlation co-efficients were transformed to the *z* statistic, setting *p* < .05 (two-sided) as the test of significance (Meng et al., 1992). Correlations less than *R* = .20 were considered weak (Cohen, 1988).

The relationship between attachment insecurity and trends in stress outcome variables that were measured longitudinally was tested using repeated-measures ANOVA. The measures that were repeated at multiple time-points and, therefore, included in this analysis were psychological distress (T1–T5), burnout-emotional exhaustion (T1–T5), burnout depersonalization (T1–T5), burnout-personal accomplishment (T1–T5), and posttraumatic symptoms (T1, T3, T5). For this analysis, participants were sorted into equal terciles of severity of attachment variables (low, medium, high). Main effects of time and attachment variables on stress outcomes were calculated, as well as the interaction between attachment variable and time, as an indicator of possible differences in temporal patterns that could suggest resilience. Comparisons that were statistically significant were investigated post-hoc to determine the nature of the difference. In these analyses, partial eta-squared was calculated as an indicator of effect size.

With respect to statistical power, for repeated measures ANOVA involving 3 groups and measurement at 5 time points, setting significance at .05, and without correcting for sphericity, a sample of 209 people has 80% power to detect an effect as small as *f* = .24 for within-group differences, *f* = .22 for between-group differences, and *f* = .27 for interactions (Repeated-Measures ANOVA, 2022). This sample size also has the power to detect correlations of *r* < .20.

## Results

There were no significant differences between participants assigned to the express or enriched surveys with respect to age, job type, gender, education, marital status, emotional exhaustion at T1, or psychological distress at T1 (data not shown). The current study includes data at all time points for all of the participants for whom attachment data is available (i.e. all 209 enriched survey participants who completed a measure of adult attachment at T4). Characteristics of these participants are presented in Table 1. Attachment anxiety and attachment avoidance were moderately correlated (correlation co-efficient = .30, *p* < .001).

**Table 1.** Characteristics of the sample.

	*N*	%
*Job type*		
Nurse	49	23.4
Other healthcare care professional	66	31.6
Non-professional with patient contact	33	15.8
Non-professional without patient contact	61	29.2
*Gender*		
Female	171	81.8
Male	33	15.8
Other/prefer not to say	5	2.4
*Ethno-cultural identity*		
African/Black	11	5.3
Asian	60	28.7
European/White	107	51.2
Hispanic	4	1.9
South Asian	14	6.7
Other	13	6.2
*Education*		
High school or less	3	1.4
College diploma	24	11.5
Undergraduate degree	63	30.1
Graduate or professional degree	119	56.9
*Marital status*		
Single	83	39.7
Married/Common-law	117	56.0
Divorced/Separated	9	4.3
*Age*		
18–30	69	33.0
31–40	54	25.8
41–50	50	23.9
51 and older	36	17.3

### Self-appraisal of personal resources

Regarding self-appraisal of resources to respond to threat, consistent with the hypothesis, we found that self-assessed occupational resilience was significantly negatively associated with both attachment avoidance and attachment anxiety, that these correlations did not differ from each other, and that the correlation of self-assessed resilience to severity of attachment insecurity was similar (Table 2, Part A). Contrary to the hypothesis, we found pandemic self-efficacy was more strongly negatively associated with attachment anxiety than with attachment avoidance. The correlation between pandemic self-efficacy and severity of attachment insecurity was similar to its association with attachment anxiety.

**Table 2.** Correlations between attachment insecurity and aspects of stress response.

Aspects of stress response (1)	Correlations with attachment insecurity				
	Attachment avoidance (2)	Attachment anxiety (3)	Significance of difference between *R*_12_ and *R*_13_		Severity of insecure attachment (4)
	*R*_12_	*R*_13_	*z*	*p*	*R*_14_
*A. Appraisal of personal resources*					
Occupational resilience	−.32***	−.30***	−0.3	.80	−.37***
Pandemic self-efficacy	−.30***	−.48***	2.4	.02	−.48***
*B. Coping behaviors*					
Denial	.07	.19**	−1.4	.15	.18*
Seeking emotional support	−.23***	.16*	−4.6	<.001	−.04
Seeking instrumental support	−.16*	.16*	−3.8	<.001	.03
Self-blame	.12	.35***	−2.8	.005	.34***
Substance use	.00	.05	−0.6	.56	.01
Active coping	−.20**	−.10	−1.2	.23	−.17*
Humor	.03	.11	−0.9	.35	.11
Religion	.03	.00	0.4	.72	.04
*C. Interpersonal problems*					
Domineering	.25***	.33***	−1.0	.32	.36***
Vindictive	.42***	.30***	1.6	.12	.44***
Cold	.51***	.30***	2.8	.005	.48***
Socially inhibited	.42***	.32***	1.3	.19	.43***
Nonassertive	.11	.29***	−2.2	.03	.24***
Exploitable	.08	.34***	−3.2	.002	.28***
Overly-nurturant	.05	.31***	−3.1	.002	.24***
Intrusive	.03	.35***	−3.9	<.001	.28***
Mean interpersonal problems	.39***	.46***	−.95	.34	.53***

*p < 0.05; **p < 0.01; ***p < 0.001.

### Coping strategies

With respect to coping (Table 2, Part B), most correlations between coping strategies and attachment measures were weak: 20 of the 24 correlations tested had correlation co-efficients weaker than .20. Exceptions were the correlations of attachment avoidance with reduced seeking of emotional support (*R* = −.23, *p* < .001) and reduced active coping (*R* = −.20, *p* < .01), the correlation of self-blame with attachment anxiety (*R *= .35, *p* < .001) and with severity of insecure attachment (*R* = .34, *p* < .001). Contrary to our hypotheses, attachment avoidance was not associated with *any* coping strategy more strongly than was attachment anxiety. The hypothesized stronger associations of attachment anxiety with seeking both instrumental and emotional support, and with self-blame were confirmed. The hypothesized association between severity of insecure attachment and coping via substance use was not confirmed.

### Interpersonal problems

Regarding interpersonal problems (Table 2, Part C), contrary to our hypothesis that attachment anxiety would be specifically associated with interpersonal problems involving excessively seeking closeness, we found attachment anxiety to be moderately strongly associated with *every* domain of interpersonal problems. Attachment avoidance was more specific in its association to problems involving excessive distancing, with an association to problems with being cold that was stronger than the association of this type of interpersonal problem with attachment anxiety. Moderately strong associations were found between attachment avoidance and being vindictive or socially inhibited, although the differences between these correlations and the corresponding correlations of interpersonal problems with attachment anxiety failed to achieve statistical significance. Attachment avoidance was not significantly associated with interpersonal problem on the affiliative side of the interpersonal circumplex.

Attachment avoidance was associated with problems related to domineering, which was not hypothesized. Attachment anxiety was more strongly associated with problem with non-assertiveness than was attachment avoidance, which was not hypothesized. Severity of insecure attachment was associated with every domain of interpersonal problems with a strength of association similar to whichever dimension of attachment insecurity had the strongest correlation.

### Stress outcomes

Three dimensions of burnout, as well as psychological distress and posttraumatic symptoms were measured longitudinally as outcomes of stressful exposure. Repeated measures ANOVA (Table 3) revealed that each outcome except personal accomplishment changed significantly over time. Furthermore, each measure of insecure attachment had a significant main effect on each stress outcome, with effect sizes ranging from .05 to .26. For each of these stress outcomes, the effect size associated with severity of attachment insecurity was greater than the corresponding effect size for attachment anxiety or attachment avoidance. These findings correspond to our hypotheses.

**Table 3.** Associations between attachment variables and changes in stress outcomes over time.

	Attachment variable			Time			Time X attachment		
	*F*	*p*	*eta^2^*	*F*	*p*	*eta^2^*	*F*	*p*	*eta^2^*
*Independent variable: attachment anxiety*									
Emotional exhaustion	12.1	<.001	.13	8.0	<.001	.05	1.4	.22	.02
Depersonalization	8.0	<.001	.12	7.6	<.001	.06	3.4	.001	.06
Personal accomplishment	6.4	.002	.10	0.8	.55	.01	1.6	.12	.03
Psychological distress	19.5	<.001	.19	11.3	<.001	.06	1.7	.09	.02
Posttraumatic symptoms	13.7	<.001	.14	3.9	.02	.02	1.1	.34	.01
*Independent variable: attachment avoidance*									
Emotional exhaustion	7.0	.001	.08	7.7	<.001	.04	0.6	.74	.01
Depersonalization	9.7	<.001	.15	6.6	<.001	.06	0.8	.58	.01
Personal accomplishment	7.9	<.001	.12	1.1	.36	.01	0.6	.74	.01
Psychological distress	7.7	<.001	.09	10.8	<.001	.06	1.4	.21	.02
Posttraumatic symptoms	4.9	.009	.05	3.5	.03	.02	1.0	.38	.01
*Independent variable: severity of attachment insecurity*									
Emotional exhaustion	19.7	<.001	.19	7.8	<.001	.05	1.0	.44	.01
Depersonalization	14.0	<.001	.20	7.5	<.001	.06	2.6	.01	.04
Personal accomplishment	12.1	<.001	.18	0.9	.46	.01	2.7	.008	.05
Psychological distress	28.3	<.001	.26	11.4	<.001	.07	2.0	.04	.02
Posttraumatic symptoms	26.8	<.001	.24	3.8	.03	.02	0.3	.85	.00

**Figure 2.**
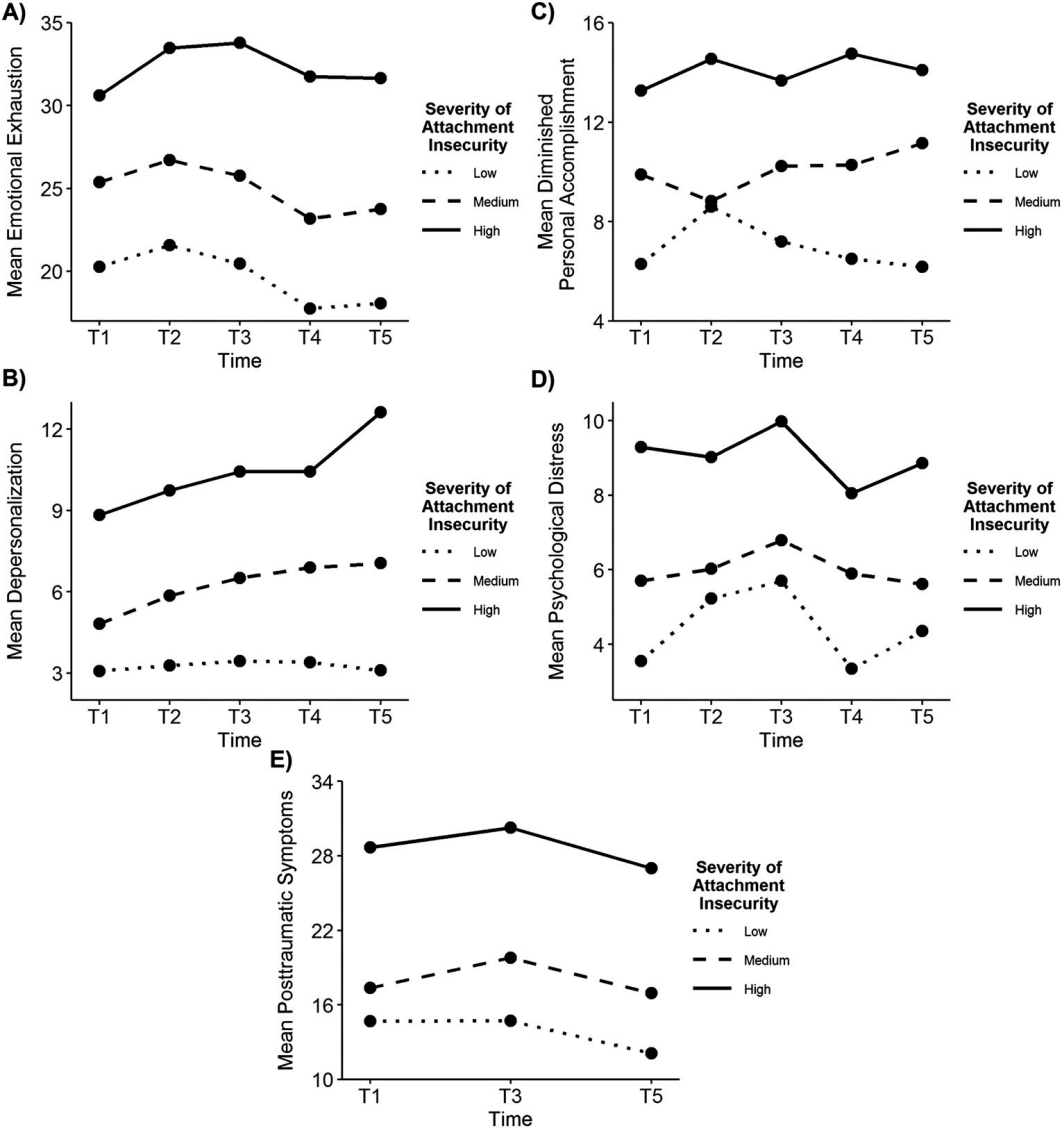
Temporal trends in psychological distress and dimensions of occupational burnout by severity of attachment insecurity.

Regarding the interaction of attachment variables and time on stress outcomes, there was a significant interaction of attachment anxiety and time with respect to depersonalization (effect size = .06), but no other significant effects of the interactions between attachment anxiety and time. There were no significant interactions of attachment avoidance and time on stress outcomes. There were significant interactions of severity of attachment insecurity and time with respect to depersonalization (effects size = .04), personal accomplishment (effect size = .05), and psychological distress (effect size = .02). Figure 2 illustrates the directionality of these relationships and the nature of the interactions. The temporal trends illustrated in Figure 2 indicate that for depersonalization, the slope of change over time is relatively flat for those with low insecurity, consistently rising over time for those with high insecurity, and rising with an intermediate slope for those with medium severity of insecurity. The slope of the curves illustrating personal accomplishment (also statistically significant) includes a trend toward increasing diminished personal accomplishment over time for those with medium or high insecurity, compared to a reduction over time (starting at T2, after an initial increase) for those with low insecurity. The trends of changes over time in psychological distress (also statistically significant) is more complex.

## Discussion

In this longitudinal study of a cohort of hospital workers for more than a year during the severe stress of a global pandemic, aspects of insecure adult attachment were significantly correlated with most aspects of stress response, especially with appraisal of one’s resources to cope, with interpersonal problems, and with stress outcomes. In particular, severity of insecure attachment was moderately strongly correlated with each measure of self-appraisal, interpersonal problems and stress outcomes. On the other hand, specificity of the relationship between dimensions of insecure adult attachment (attachment anxiety or attachment avoidance) and particular aspects of stress response was not strongly supported. Only seven of fourteen hypotheses of dimensional specificity were confirmed, which were the relationships of attachment anxiety to seeking instrumental support, seeking emotional support, and self-blame, the association of attachment avoidance with interpersonal problems related to being cold, and the associations of attachment anxiety to interpersonal problems related to being exploitable, overly nurturant, and intrusive.

The lack of support for several hypotheses of dimensional specificity with respect to attachment anxiety and attachment avoidance suggests that in the context we studied, overall severity of the experience of insecurity was more relevant than the specific interpersonal and affect-regulation strategies (hyper-activating or de-activating) used to manage this experience. This differs from several earlier studies reviewed above. The reason for this difference may relate to the nature of the stressful situation since it is unusual in the attachment literature to study the impact of a relatively severe environmental threat over many months. In particular, a severe and persistent threat may overwhelm de-activating strategies, such that perceived (‘felt’) insecurity is similar in those with different attachment patterns. In such a case, the severity of insecurity could be a better predictor of outcomes than the type of insecurity. It is also possible that this group of hospital workers (consisting of >50% healthcare professionals, >80% women and >85% university graduates) differs from other populations studied.

Most correlations between measures of attachment and coping strategies were weak. This may be because coping strategies are specific to their context and, therefore, are dynamic in a pandemic which presents changing challenges and demands over time. It is noteworthy in this respect that coping was measured at T1, in the fall of 2020, during a steady increase in cases leading up to a province-wide lockdown, which accompanied the pandemic’s large second wave in this region. In this context, attachment anxiety was specifically associated with seeking support, both emotional and instrumental, and with self-blame. Severity of attachment insecurity was significantly associated with ineffective coping strategies: self-blame, denial, and not employing active coping.

Correlations of attachment measures with seeking emotional or instrumental support illustrate a property of severity of attachment insecurity that follows from it being calculated as the sum of the vectors of attachment anxiety and attachment avoidance: when the relationships of attachment anxiety and attachment avoidance with another variable are in opposite directions (i.e. one positive and the other negative), the correlation of severity of insecurity with that variable is very weak. On the other hand, when the relationships of attachment anxiety and attachment avoidance with another variable are in the same direction but differ in strength, severity of attachment insecurity is a good estimate of the stronger association.

Interpersonal problems are closely theoretically related to dimensions of insecure attachment because the definitions of attachment anxiety and attachment avoidance consist, in part, of hyper-activating and deactivating interpersonal strategies which can lead to problems. The theoretically predicted relationships between specific dimensions of insecurity were confirmed for problems being cold (attachment avoidance) and problems with being exploitable, overly-nurturant, or intrusive (attachment anxiety). Although the association between attachment avoidance and problems with being vindictive or socially inhibited were moderately strong (>.40) as predicted, these associations were not significantly stronger than the association of attachment anxiety to these types of interpersonal problems because the associations with attachment anxiety were stronger than expected. Indeed, attachment anxiety was moderately strongly associated with *every* type of interpersonal problem. The latter observation may indicate globally problematic interpersonal relationships, or might suggest a bias towards endorsing problems of all types among those who report a hyper-signaling interpersonal style.

Each measure of attachment insecurity had a significant main effect on every measure of stress outcome. That the largest effect sizes in each case were associated with severity of attachment insecurity is consistent with our hypotheses and suggests that severity of attachment insecurity may be a useful single measure summary of attachment status in stress studies, especially in circumstances in which parallel analyses with separate dimensions of insecurity are inconvenient.

Severity of insecurity was related to differences in changes over time in two dimensions of occupational burnout (depersonalization and personal accomplishment), as well as psychological distress. Inspection of the difference in the direction of change over time in hospital workers with high vs. low severity of attachment insecurity (Figure 2), suggests that for those with low severity of attachment insecurity (i.e. the more secure members of the cohort) depersonalization was relatively low and not increasing over time, whereas for those with high severity of attachment insecurity, depersonalization was high and rising as the pandemic progressed. This difference suggests that low severity of attachment insecurity is associated with resilience (i.e. that it is protective with respect to the depersonalization effects of occupational burnout). Similarly, those with low severity of attachment insecurity reported, on average, an initial increase in their sense of diminished personal accomplishment from T1 to T2, followed by recovery with improvements at each time point from T2 to T5. In contrast, those with high severity of insecure attachment reported a relatively lower appraisal of personal accomplishment which did not improve over time. This contrast is also consistent with greater attachment security being associated with resilience. With regard to psychological distress, although the main effect of higher severity of insecurity being associated with higher psychological distress is clear, differences in temporal changes by severity of attachment insecurity are harder to interpret.

The interpretation that attachment security was associated with resilience with respect to depersonalization and personal accomplishment depends on the assumption that patterns of adult attachment were stable over the period of this study. This assumption is consistent with the construct of adult attachment, which theoretically describes relatively stable traits (Mikulincer & Shaver, 2016). Empirically, test-retest stability of self-report measures of adult attachment measured with the ECR has supported high stability over periods of 2–3 months (Picardi et al., 2011; Smith et al., 1999) and similar measures have found high stability over 1–2 years (Mikulincer & Shaver, 2016). While patterns of attachment are thought to adapt to life circumstances, changes in attachment patterns related to major life events have typically been observed over periods of many years (Weinfield et al., 2000). Thus, while it is a limitation that attachment was measured at T4 rather than at baseline, and this measure was not repeated to confirm stability over time, both theory and prior evidence suggest that attachment measures are unlikely to have changed during the study.

The strengths of this study include its repeated prospective measurement of aspects of stress response using validated measures, high retention of participants over time, and exposure to a severe and prolonged environmental stress. Multiple longitudinal measures of stress outcomes allow a direct test of resilience, understood as a better course of stress outcomes over time. The sample size was sufficient to detect small to moderate between-group, within-group and interaction effects in repeated measures ANOVA and weak correlations (*R* < .20). There are also important limitations. All measures depend on self-report and there is no corroboration of findings with objective or observation–based measures. We assume that adult attachment is a stable trait over time in our interpretation of results, but this assumption is not tested with repeated measures of attachment. Constructs were measured at different time points which may reduce the strength of association between variables that vary over time. The calculation of the vector of severity of attachment insecurity assumes that attachment anxiety and attachment avoidance are independent (correlation = 0, and therefore, form two right-angled sides of a triangle), whereas empirically they are weakly related. Since *R* = .30, each attachment dimension actually explains about 9% of variance in the other. It is also noteworthy that our test of multiple hypotheses used a standard of significance of *p* < .05. Since the independent variables in these analyses are somewhat correlated, and the dependent variables are also somewhat correlated (data not shown), a Bonferroni correction would be overly conservative. Nonetheless, it is noteworthy that if we had applied the Bonferroni correction to our analyses, almost all of the findings reported as significant, would have remained significant under the more stringent criterion (coping: 8 comparisons, *p* < .006; interpersonal problems: 9 comparisons, *p* < .006; stress outcomes, 5 comparisons, *p* < .01).

In conclusion, we find evidence supporting the association of adult attachment insecurity with many aspects of the stress response and its outcomes in a context that has received little or no prior study: working in healthcare for over a year during an extraordinarily stressful global pandemic. We introduce a measure of overall severity of attachment insecurity that performs well as a summary measure of attachment insecurity in this context (with the specific exception of associations in which attachment anxiety and attachment avoidance are related with a variable in opposite directions, as they were for seeking support). Furthermore, using longitudinal data over five measurements, we find evidence that for the depersonalization and personal accomplishment dimensions of occupational burnout, lower severity of insecure attachment is associated with resilience. Further research is encouraged to determine the characteristics of this measure of severity of attachment insecurity in other contexts.
